# Herbal Medicine for Hot Flushes Induced by Endocrine Therapy in Women with Breast Cancer: A Systematic Review and Meta-Analysis

**DOI:** 10.1155/2016/1327251

**Published:** 2016-06-21

**Authors:** Yuanqing Li, Xiaoshu Zhu, Alan Bensussan, Pingping Li, Eugene Moylan, Geoff Delaney, Luke McPherson

**Affiliations:** ^1^Key Laboratory of Carcinogenesis and Translational Research (Ministry of Education), Integrative Department of Traditional Chinese Medicine with Western Medicine, Peking University School of Oncology, Beijing Cancer Hospital & Institute, Beijing 100142, China; ^2^National Institute of Complementary Medicine (NICM), Western Sydney University, Macarthur, NSW 2751, Australia; ^3^Liverpool Cancer Center, Liverpool Hospital, Liverpool, NSW 2170, Australia

## Abstract

*Objective.* This systematic review was conducted to evaluate the clinical effectiveness and safety of herbal medicine (HM) as an alternative management for hot flushes induced by endocrine therapy in breast cancer patients.* Methods.* Key English and Chinese language databases were searched from inception to July 2015. Randomized Controlled Trials (RCTs) evaluating the effects of HM on hot flushes induced by endocrine therapy in women with breast cancer were retrieved. We conducted data collection and analysis in accordance with the Cochrane Handbook for Systematic Reviews of Interventions. Statistical analysis was performed with the software (Review Manager 5.3).* Results.* 19 articles were selected from the articles retrieved, and 5 articles met the inclusion criteria for analysis. Some included individual studies showed that HM can relieve hot flushes as well as other menopausal symptoms induced by endocrine therapy among women with breast cancer and improve the quality of life. There are minor side effects related to HM which are well tolerated.* Conclusion.* Given the small number of included studies and relatively poor methodological quality, there is insufficient evidence to draw positive conclusions regarding the objective benefit of HM. Additional high quality studies are needed with more rigorous methodological approach to answer this question.

## 1. Introduction

As a result of increased screening and improved treatment, more women are becoming long-term survivors with breast cancer. This increasing survivorship has increased demand to improve quality of life (QoL). Endocrine therapy, is a widely used strategy in improving breast cancer survival [1]. Hot flushes are the most frequently reported adverse effects (AEs) associated with endocrine therapy in breast cancer. The symptoms are sometimes so frequent and severe that they interfere with daily activities and decrease QoL of breast cancer survivors [2, 3]. Hot flushes are described as a sudden episode of a sensation of internal heat often preceded by chills and redness of face and upper body and are often accompanied by profuse sweating and dizziness [4]. The incidence of hot flushes reported was approximately 39.9%–54% with tamoxifen, 37% with initial aromatase inhibitors (AIs) therapy, and 42%–48% with sequential AI therapy following 2-3 years of tamoxifen [5–8]. Hot flushes are most commonly experienced in the first year after commencing adjuvant therapy and gradually reduce over time following breast cancer treatment, particularly following the cessation of tamoxifen [9]. The exact etiology of hot flushes is unknown. The sudden decline in oestrogen levels [10] has one reason, but there is not sufficient evidence to explain their occurrence in breast cancer patients with endocrine therapy. It may be also related to chemotherapy-induced ovarian disruption and another is the natural aging process which is similar to that experienced by healthy women [11].

There are many pharmacological therapies that currently treat hot flushes. It is reported that hormone replacement therapy (HRT) is considered to be effective for hot flushes; however it raised significant concern when it is used on women with breast cancer [12, 13]. Animal and in vitro models have shown that progestational agents may increase or accelerate breast cancer development; this is not thought to be an appropriate intervention [14]. Tibolone was confirmed to create a significant reduction in hot flushes and improvement of QoL in breast cancer patients but at the cost of increased risk of breast cancer recurrence [15]. In addition to clonidine and the anticonvulsant gabapentin, several selective serotonin reuptake inhibitors (SSRIs), such as venlafaxine, paroxetine, fluoxetine, and citalopram, were reported to alleviate hot flushes [16–21]. Unfortunately, using those agents is limited by their AEs profile, which includes dry mouth, decreased appetite, nausea, constipation, and drowsiness, and contributed to increased rate of participant dropout in study [22]. Furthermore, aforementioned antidepressant drugs used in conjunction with tamoxifen could affect anticancer efficacy of endocrine therapy and increase the risk of recurrence of breast cancer due to inhibition of cytochrome P450 2D6 (CYP2D6) and by reducing the active metabolite of tamoxifen-endoxifen plasma concentrations [23–25]. As a popular complementary and alternative medicine, vitamin E was reported to decrease frequency of hot flushes. This was statistically but not clinically significant [26, 27].

HMs are widely used for reduction of various AEs related to chemotherapy, radiotherapy, and endocrine therapy in breast cancer patients. HMs include herbs, herbal materials, herbal preparations, and finished herbal products, which contain parts of plants, plant materials, or combinations as active ingredients [28]. Traditional use of HMs refers to the long historical use of these medicines [28]. Their use is well established and widely acknowledged to be safe and effective. There are numerous studies investigating HMs for hot flushes and menopausal symptoms in normal women. Studies on some extracts with the isoflavones from HM were found to significantly reduce mean daily hot flush frequency compared with placebo [29]. However, there are other HM studies that failed to demonstrate a significant improvement in hot flushes symptoms [30, 31]. There are other HMs used in many countries that have potential effectiveness in decreasing hot flushes or menopausal symptoms, such as hop (*Humulus lupulus*) [32], linseed or flaxseed [33], maritime pine bark extract [34], maca (*Lepidium meyenii*) [35], and* Hypericum perforatum* L. (St. John's Wort) [36]. While these are effective in reducing hot flushes in women without breast cancer, there is no evidence that the aforementioned HMs are safe and effective treatment of hot flushes induced by endocrine therapy in breast cancer. As for Chinese herbal formulations, a RCT in a non-breast cancer setting conducted by a Dutch research team [37] used Zhi Bai Di Huang Wan versus HRT and placebo to treat menopausal symptoms. It was concluded that the formula was more effective in reducing the amount of hot flushes compared to placebo.

There is growing interest in HM for hot flushes induced by endocrine therapy in women with breast cancer; this has led to an increased number of clinical trials being performed in this area. However, specific evidence-based recommendations on the use of these herbs in breast cancer patients cannot be made. There are some reviews that have explored the effect and safety of HM for vasomotor symptoms including hot flushes and accompanying symptoms in women with early breast cancer. There are also a number of studies that have explored the effect and safety of soy and some food products [38]. Some reviews, regarding the efficacy of herbal treatments for hot flushes, have not been conducted in women with breast cancer and many are not specifically related to endocrine therapy [39]. Some reviews have had a focus on the safety of herbal medicinal products in women with breast cancer but not the effect of herbal medicines on hot flushes [40–42]. Therefore, we conducted this systematic review to determine whether HM is effective and safe for reducing hot flushes and vasomotor symptoms, induced by endocrine therapy in patients with breast cancer, and to identify the limitations of existing studies as a guide for future clinical research in this area. If shown to be effective, HM can offer an alternative intervention to those patients.

## 2. Methods

### 2.1. Search Strategy

**Figure 1:**
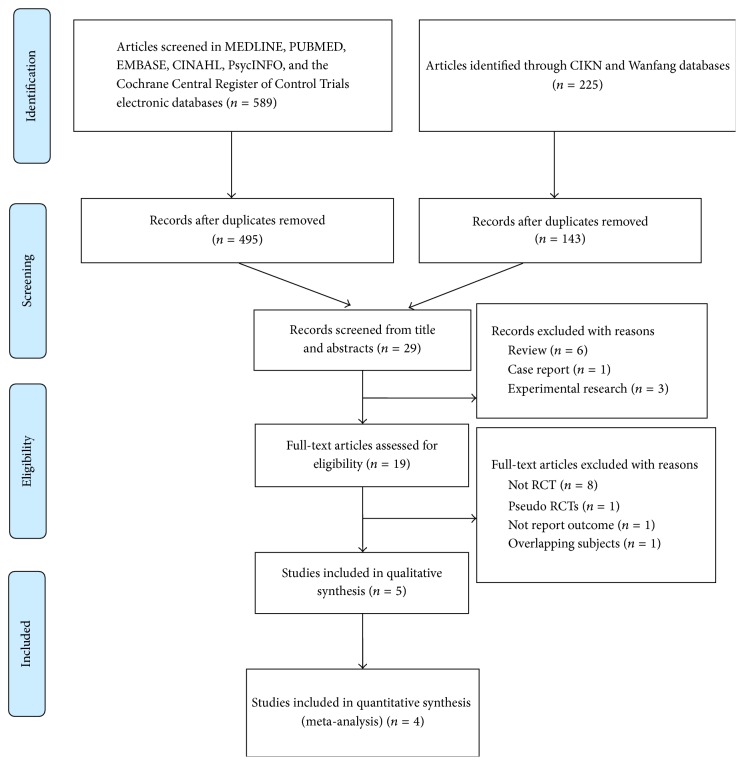
Flowchart of article search.

The search of the scientific literature was performed in the English language databases including MEDLINE, PubMed, EMBASE, PsycINFO, CINAHL, and the Cochrane Central Register of Control Trials (CENTRAL) electronic databases. The search for scientific literature was also performed in Chinese language databases including China National Knowledge Infrastructure (CNKI) and Wanfang databases. We used search strategies with the following medical subject headings (MeSH): “Chinese herbal medicine”, or “traditional Chinese medicine”, or “herbal medicine”, or “plants medicine”, or “kampo medicine”, and “hot flushes”, or “hot flashes”, or “menopausal symptoms”, or “vasomotor symptoms”, in combination with “breast cancer”. There are limited articles included in the search MeSH as “endocrine therapy”. Therefore we selected articles related to endocrine therapy after completion of the above search strategy (date last searched: 24 July 2015).  Figure 1 shows the whole flowchart of article search.

### 2.2. Selection Criteria

We included only RCTs that tested the effectiveness of HM for hot flushes induced by endocrine therapy in female breast cancer patients. The inclusion criteria were controlled studies where women were diagnosed with breast cancer and were treated with endocrine therapy. We included any types of HM interventions managing hot flushes. Detailed preparations may be single herbs, extract ingredient from one or several herbs, and Chinese herbal formulae such as standardised formula and tailored formula. Control interventions may include placebo, conventional therapy (e.g., HRT), western medicine therapy (e.g., venlafaxine), acupuncture or other complementary therapies (e.g., yoga), and no intervention for hot flushes or other HM but not for hot flushes. Quasi-RCTs, case reports, studies including fewer than 10 participants, experimental studies, incomplete reports studies, and review studies were excluded. We also excluded dietary products such as soy products. Two reviewers (Yuanqing Li and Luke McPherson) independently screened articles to identify those that met the study criteria. Yuanqing Li screened title and abstract of articles found in the search and discarded trials that were clearly ineligible. Two authors (Yuanqing Li and Xiaoshu Zhu) assessed whether the trials met the inclusion criteria, with disagreements resolved by discussion. When articles contained insufficient information to make a decision about eligibility, we attempted to contact authors of the original reports to obtain further details.

### 2.3. Data Collection and Analysis

We conducted data collection and analysis in accordance with the Cochrane Hand book for Systematic Reviews of Interventions [48]. Two review authors (Yuanqing Li and Xiaoshu Zhu) independently extracted data using a form designed by the review authors for this purpose. For each included trial, we collected information regarding the location of the trial, methods of the trial, risk of bias, participants (age range and eligibility criteria), type of interventions, and effect of interventions. We assessed risk of bias using The Cochrane Collaboration's “Risk of Bias” tool. For each study, the seven domain-based criteria were as follows: random sequence generation, quality of allocation concealment, blinding of participants and personnel, blinding of outcome assessors, completeness of outcome data, risk of selective outcome reporting, and other potential bias. The review authors assessed each domain as at high, low, or unclear risk of bias. The following comparisons were included: ① HM versus no treatment; ② HM versus placebo; ③ HM versus active medications; ④ HM versus HT; ⑤ HM versus other complementary therapy.

### 2.4. Statistical Analysis

Statistical analysis was performed with the software (Review Manager 5.3) for meta-analysis. In studies that reported the exact same outcomes of continuous data, the mean difference (MD) was calculated between treatment groups. If similar outcomes were reported on different scales, the standardized mean difference (SMD) was calculated. We combined data from included studies using fixed-effects models. We presented 95% confidence intervals (CI) for all estimates. Dichotomous outcomes were analysed as per woman randomized (e.g., number of women with an adverse effect/total number of women randomized). Statistical heterogeneity among studies was evaluated using the Cochran's *Q* and *I* ^2^ statistics [49]. Heterogeneity was considered present for *P* < 0.05 or *I* ^2^≧50%.

## 3. Results

### 3.1. Study Characteristics

**Figure 2:**
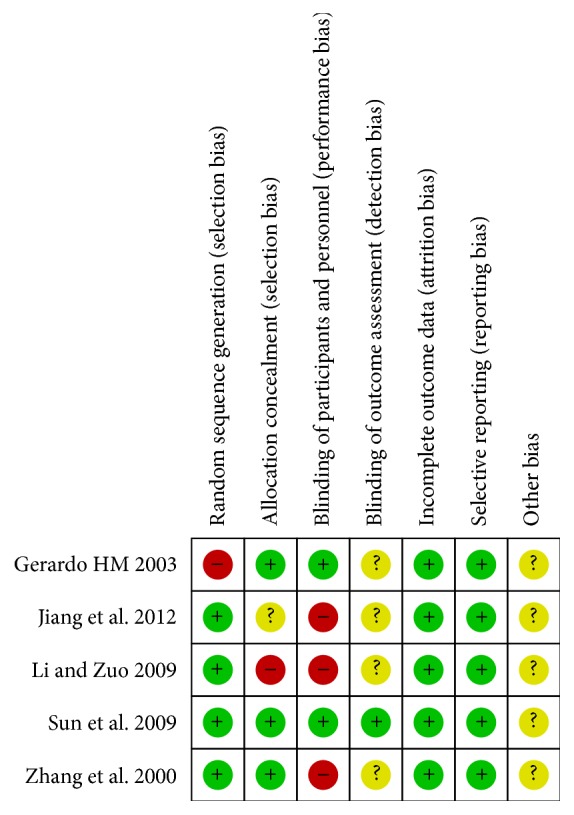
Figure to demonstrate the bias with eligible studies as noted by authors. For each study, the presence (+) and absence (−) of a characteristic are recorded. If the characteristic was not clear in the trial, then it was marked as uncertain (?).

A total of 814 articles were retrieved from electronic databases, 275 from PubMed, 252 from EMBASE, 36 from MEDLINE EBSCO, 17 from CINAHL EBSCO, 6 from PsycINFO EBSCO, 3 from the Cochrane Library, 143 from CNKI, and 82 from Wanfang databases. After checking duplicates, 638 articles remained. Articles not related to endocrine therapy were excluded upon review of the title and abstracts. This process left 19 articles that were eligible for the inclusion criteria. Because we did not use “endocrine therapy” as a MeSH, the difference of article number between initial search and reviewed search is significant. 7 articles that were not RTCs were further excluded after full texts were reviewed. One study was considered as Pseudo RCT. One study did not have the required information to calculate results. One article was excluded due to overlapping subjects. One study was excluded pertaining to low quality after assessment [50]. Therefore the final analysis involved five articles [43–47]. According to the bias judgement of Review Manager analysis (Figure 2), one study was high quality [46] and other studies were moderate bias risk. Only one study [46] in the included studies has a double-blind design.

The number of participants varies from 60 to 136. The total number of participants included in the studies for this analysis amounts to 397. Of the five studies, two studies evaluated HM in comparison to no treatment [43, 45], one study compared the effects of HM with placebo [46], and two studies examined the effects of HM versus HM [44, 47].  Table 1 presents basic characteristics of all five trials that compared HM preparations (monotherapy or combination therapies) with placebo (the authors, published year, age span, sample size, outcomes, and intervention method).

### 3.2. Summary Analysis of the Included Studies

There are a total of five studies being analysed. The outcomes of analysis for the primary and secondary endpoints only involved the following 3 categories.

#### 3.2.1. HM versus No Treatment

This analysis includes two studies, of which the results are concerned with the issue of overall scores of menopausal symptoms.

In the study by Li and Zuo [45], the change of overall scores of menopausal symptoms showed significant difference in the Kupperman Index (KI), which is used worldwide to investigate menopausal symptoms including hot flushes. Compared with control group, MD is 13.36 [95% CI 9.37–17.35]. After 2 months the scale of KI decreased from an initial 43.41 ± 7.369 to 30.43 ± 8.905 in the treatment group and from 43.82 ± 7.222 to 43.79 ± 7.32 in the control group (*P* < 0.01). The baselines showed no significant differences.

In the study by Hernández Muñoz and Pluchino [43], evaluation of menopausal symptoms was assessed with frequency of hot flushes episodes combined with sweating and sleep disturbance. Hot flushes were considered severe when five or more sudden episodes of heat are experienced during the day, accompanied by sweating, sleep disturbances, and feeling of irritation and anxiety. Less than five episodes of heat with discrete sweating were classified as moderate hot flushes. The difference between values of numbers of hot flushes was not significant at 6 months, either for severe or for moderate hot flushes (5–9% decline; *P* = 0.71), but they were significantly different at the end of the study (after 12 months). Among the 90 study participants included in the intervention group 46.7% were free of hot flushes while none were free among the usual-care group. Severe symptoms cases were 24.4% versus 73.9% with odds ratio (OR) 0.11 [0.05, 0.26].

**Figure 3:**
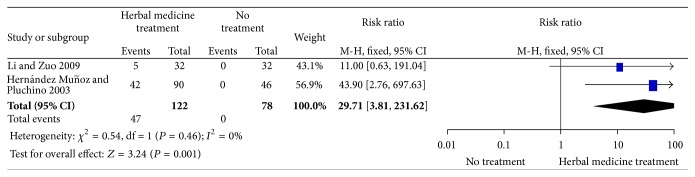
Effect of HM and no treatment on risk ratio in those free of menopausal symptoms. Forest plot includes only two studies.

The dichotomous data in these two studies assessing those free of menopausal symptoms were suitable for meta-analysis. There was a difference between HM group and the no treatment group, in favour of the HM group (RR 29.71; 95% CI 3.81–231.62; 2 RCTs, 200 women), with no heterogeneity (*I* ^2^ = 0%), Figure 3. 

*Adverse Events*. There were no significant AEs in one study [45]. In another study [43], eleven minor AEs occurred: seven in the usual-care group and four in the intervention group. No serious events were reported. One study [45] reported that there was no significant difference between two groups in hormone levels with OR 1.02 [95% CI 0.46–2.24]. This study also compared the thickness of endometrium, which showed increase in both groups, but there was no significant difference (*P* = 0.14) with MD 0.80 [95% CI 0.57, 1.03].

#### 3.2.2. HM versus Placebo

Only one study was included. The study of Sun et al. [46] reported decrease of the frequency of hot flushes in two groups; OR for traditional Chinese medicine (TCM) called Shugan-Liangxue Compound versus placebo was 3.12 [95% CI 1.13, 8.60]. In TCM group, 15.2% were free of hot flushes and 42.4% showed no change, while no participant was free of hot flushes and 69.7% showed no change in placebo group. The difference between values was significant (*P* = 0.012).

Sleeping behaviour was studied in this publication. The ratio of insomnia improvement RR was 2.69 [95% CI 1.00, 7.28] (*P* = 0.05). 

*Adverse Events*. There were no significant side effects in all participants.

#### 3.2.3. HM versus HM

In this profile two studies used different scales for evaluating symptoms, which made a quantitative comparison to hot flushes impossible.

In the study by Jiang et al. [44], a significant reduction of 1.47 points in overall score of KI for menopausal symptoms was observed in TCM group (kidney-reinforcing and liver-regulating formula) with 95% CI = 0.9–2.05 (*P* < 0.01). In this study, TCM symptoms score also was observed with a significant reduction of 2.27 points with 95% CI = 1.39–3.15 (*P* < 0.01).

In the study by Zhang et al. [47], the incidence of hot flushes was evaluated. This value in observation group decreased from 66.7% to 33.3%, while it decreased from 70.0% to 60.0% in control group. OR was 0.33 [95% CI 0.12, 0.96]. Study also evaluated other menopausal symptoms for one month, there was significant improvement in the subscales including night sweat (*P* = 0.0017), insomnia (*P* = 0.029), irregular menstruation (*P* = 0.0017), palpitation (*P* = 0.003), and heat sensation in chest, palms, and soles (*P* = 0.004).

**Figure 4:**
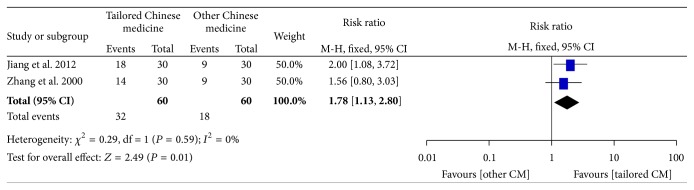
Effect of HM and no treatment in QoL. Forest plot includes only two studies.

Both studies showed positive effect on QoL. The pooled RR estimate in QoL showed significant improvement in the tailored TCM group which means formulas were prescribed guided by TCM discipline. RR estimate was 1.78 [95% CI 1.13–2.80], which suggests that tailored TCM group showed a great improvement of QoL than other TCM groups. There was no significant heterogeneity with *χ* ^2^ = 0.29 (df =1, *P* = 0.59) and *I* ^2^ = 0%. These results are depicted in Figure 4.

*Adverse Events*. In the studies by Jiang et al. [44], the articles reported that the changes of hormones level were not significantly different between the two groups.

## 4. Discussion

This review summarizes the evidence from RCTs of mono- or combined use of HM for hot flushes induced by endocrine therapy in women with breast cancer. It is the first meta-analysis to review HM alone in treatment of hot flushes induced by endocrine therapy and first to evaluate the methodological quality of existing RCTs on this crucial issue.

Based on the existing data, there is no clear evidence of the benefit of HM in treatment of hot flushes induced by endocrine therapy in women with breast cancer, although some individual studies showed slight improvements. However, there is a beneficial effect of HM on menopausal symptoms and improvement of QoL to some extent. This review focused on hot flushes and menopausal symptoms; both individual studies and the main meta-analysis in HM versus no treatment profile showed a favourable result. This result was also found in the individual study in HM versus placebo. We also found that TCM was more effective when guided by a Chinese medical discipline. Therefore, reasonable HM may be considered as an alternative treatment in treating hot flushes induced by endocrine therapy among women with breast cancer, especially for whose worried about the adverse effects from HRT or other nonhormonal therapies. Nevertheless, this assumption needs to be supported by more extensive, high quality, and transparent studies with an appropriate number of subjects. More randomized, double-blind, multicenter clinical trials that are designed with rigorous methodology are required to draw firm conclusions.

The strength of this systematic and meta-analysis review is the investigation of a uniform population. All participants in this review are patients suffering from breast cancer. Patients with hot flushes induced by endocrine therapy were included, while menopausal symptoms induced by chemotherapy or radiotherapy were excluded. Only studies without obvious risk of bias were included in this review. There were many other studies of HMs and TCM formulas related to hot flushes or menopausal symptoms. We also excluded studies about soy isoflavone products which were considered as food and health products.

There are some potential limitations of the meta-analysis results. The results showed slight improvement on the total effect of menopausal symptoms and hot flushes as well as other simultaneous symptoms, while the overall effect should be interpreted very carefully because the analyses were based on a small number of included studies. In those five included studies, there are no multicenter and large size trials. The total sample size is only 397 which is too small for a meta-analysis. The main reasons for the limited number of articles included in this review are the following: (1) Clinical trials on hot flushes related to endocrine therapy are still insufficient due to failure to report adverse effects. (2) Given the small number of eligible trials, we excluded studies with biased evaluation and low quality literature. On the other hand, varying methodological quality of individual trials and lack of standardized measurement of hot flushes symptom scores in all trials make difficult it to conduct a meta-analysis. In those five studies, three studies used KI but no hot flushes diary. The results only showed the overall score of menopausal symptoms. One study used hot flushes diary and assessed vasomotor symptoms. Results only reported total effective rates of vasomotor symptoms but were not specifically tailored to the effect of hot flushes. One study used hot flushes diary to evaluate frequency and severity of hot flushes and disturbance of sleep, but there are no adequate mean difference (MD) variable extracted from data. Therefore, there is little heterogeneity among studies in different profiles, making the comparison of the studies difficult or impossible. Thirdly, there were no profound reports on the effect on hot flushes, as well as night sweat which is the main accompanying symptoms. The lack of transparency and deficient information made the interpretation of studies difficult. In addition, the period of observation and follow-up was too short to assess advantage and disadvantage of HM. Although the observation period in one study was for 12 months, it was within 3 months in other four studies. It is important to note that all included studies reported a favorable effect in hot flushes, but only one study reported minor AE. Reports of AE were too brief from study to study and usually relied on self-reported symptoms experienced in the course of the trial. For long-term adverse effects the reports were limited by too short an observation and follow-up period.

We should also consider the effects of phytoestrogen. Some herbs were reported to act by enhancing oestrogen production or have oestrogen-like effects, such as Dang Gui [51] and Ren Shen (Radix Panax ginseng) [52]. It is considered that phytoestrogens may stimulate breast cancer and decrease the antitumor effects of tamoxifen. As the literature is conflicting and the safety of phytoestrogens in breast cancer patients is unknown, it has been suggested that high-dose phytoestrogen supplement should not be recommended to these women [53]. As a result, data on AEs are not definite enough for us to draw any conclusions and safety needs to be assessed particularly regarding potential herb-drug and herb-herb interactions and long-term AEs.

In this review, we have not included other language databases especially Japanese and Korean databases, so we considered it was possible that some studies related to HM for hot flushes induced by endocrine therapy in patients with breast cancer have not been searched and included. However, for reducing the potential bias during analysis, every step in conducting this review has been done by two authors individually; if there is some difference we discussed the problem to reach a consensus. Furthermore, we need to think of potential sources of heterogeneity including preparation type (mono or multiple, a standard herbal formula, or tailored formula) and dosage.

In conclusion, based on the overall results of the available studies, we could not confirm the positive effects of HM on hot flushes and quality of life induced by endocrine therapy in women with breast cancer. We need more studies with higher quality data to assess the effects on hot flushes and other menopausal symptoms, over the long-term, as well as a more comprehensive evaluation of adverse effects associated with HM.

## Figures and Tables

**Table 1:** Characteristics of 5 articles.

Author (year)	Country	*n*	Age	Comparison and sample size	Endocrine therapy	Intervention (usage, dosage, and duration)	Treatment duration	Outcomes and measurement	Design	Results
Hernández Muñoz and Pluchino 2003 [43]	Venezuela	136	35–52	(i) Intervention: *Cimicifuga racemosa* (*n* = 90) (ii) Control: no treatment group (*n* = 46)	TAM	(i) CR BNO 1055 (one tablet corresponds to 20 mg of herbal drug *Cimicifuga racemosa*), one tablet twice daily (bid), 12 months (ii) No treatment	12 months	(i) Frequency & intensity of hot flushes measured by a diary and menopausal symptoms (ii) Questionnaire (iii) AEs measured by a self-report of events	Two-armed, randomised, and open study	(i) Frequency and severity of hot flushes were reduced after intervention (ii) Minor AEs events were reported

Jiang et al. 2012 [44]	China	60	46.60 ± 5.51 versus 44.57 ± 6.99	(i) Intervention: CHM Yishen Tiaogan Decoction (*n* = 30) (ii) Control: CHM Gengnianan (*n* = 30)	Not mentioned	(i) kidney-reinforcing and liver-regulating formula bid (containing Shendi, Shanyao, Shanyurou, Danpi, Baihe, Baishao, Yujin, Foshou, Fuxiaomai, Muli, Nvzhenzi, Gancao, and Hanliancao) (ii) Gengnian An capsule 0.3 g tid (containing Shenshudi, Zexie, Maidong, Yuanshen, Fuxiaomai, Danpi, Fuling, Zhenzhumu, Xianmao, Wuweizi, Cishi, Shouwuteng, Gouteng, and Zhishouwu)	8 weeks	(i) Overall menopausal symptoms scores measured by reduction rate of Kupperman Index (KI) scores (ii) Successful rate (iii) Quality of life (QoL) measured by Karnofsky scores (KPS) (iv) Hormone profile measured by hormone levels in serum (v) AEs measured by full blood counts and liver and renal functions in serum and ECG	Two-armed, randomised, and open study	(i) The main clinical symptoms were ameliorated after treatment, especially (ii) The hectic fever and sweating and irritability

Li and Zuo 2009 [45]	China	64	35.23 ± 5.33 versus 36.86 ± 4.98	(i) Intervention: CHM Zhibo Dihuang Wan (*n* = 32) (ii) Control: no treatment (*n* = 32)	TAM	(i) Zhibai Dihuang Wan (containing Zhimu, Huangbai, Dihuang, Shanyurou, Danpi, Fuling, Zexie, and Shanyao) 8 wan (3 g) tid (ii) No treatment	2 months	(i) Overall menopausal symptoms scores measured by reduction rate of KI (ii) Hormone profile measured by E2 and FSH in serum (iii) Endometrium thickness measured by ultrasound scan (iv) AEs measured by liver and renal functions, urine routine test, and full blood counts	Randomised single-blind study	(i) Improvements on flush, perspiration, insomnia, fatigue, and irritation, without obvious side effects

Sun et al. 2009 [46]	China	73	45.9 ± 5.1 versus 46.4 ± 4.1	(i) Intervention: CHM Shugan Liangxue Decoction (*n* = 37) (ii) Control: placebo (*n* = 36)	TAM	(i) Shugan-Liangxue compound (containing Chaihu, Danpi, Baiwei, Baishao, Wuweizi, etc.) 100 mL daily (ii) Placebo (Shanzha, bitter flavor)	3 weeks	(i) Frequency and severity of hot flushes measured by KI (ii) Sleep quality measured by a self-reported diary (iii) AEs measured by a self-report, full blood counts, liver and renal function, and hormone levels	Double-blind, randomised placebo-controlled study	(i) Effective in alleviating hot flashes and mi proving the condition of sleep

Zhang et al. 2000 [47]	China	60	35–70	(i) Intervention: CHM Ruxian 1# (*n* = 30) (ii) Control: CHM Ruxian 2# (*n* = 30)	TAM	(i) Formula based on principle of regulating liver Qi and tonifying kidney Yin, combined with formula based on principle of clearing heat and toxin (ii) Formula based on principle of clearing heat and toxin	30 days	(i) Hot flushes and other menopausal symptoms measured by indefinable tool (ii) QoL measured by KPS	Randomised, parallel study	(i) Symptoms such as flush, insomnia, night sweat, palpitation, depression, and heat sensation in the chest, palms, and soles were improved and QoL changed significantly
